# A Decision Analytic Approach to Exposure-Based Chemical Prioritization

**DOI:** 10.1371/journal.pone.0070911

**Published:** 2013-08-05

**Authors:** Jade Mitchell, Nicolas Pabon, Zachary A. Collier, Peter P. Egeghy, Elaine Cohen-Hubal, Igor Linkov, Daniel A. Vallero

**Affiliations:** 1 Biosystems & Agricultural Engineering, Michigan State University, East Lansing, Michigan, United States of America; 2 Physics Department, Carnegie Mellon University, Pittsburgh, Pennsylvania, United States of America; 3 Environmental Laboratory, Engineer Research and Development Center, United States Army Corps of Engineers, Concord, Massachusetts, United States of America; 4 Office of Research and Development, United States Environmental Protection Agency, Research Triangle Park, North Carolina, United States of America; National Institute of Environmental and Health Sciences, United States of America

## Abstract

The manufacture of novel synthetic chemicals has increased in volume and variety, but often the environmental and health risks are not fully understood in terms of toxicity and, in particular, exposure. While efforts to assess risks have generally been effective when sufficient data are available, the hazard and exposure data necessary to assess risks adequately are unavailable for the vast majority of chemicals in commerce. The US Environmental Protection Agency has initiated the ExpoCast Program to develop tools for rapid chemical evaluation based on potential for exposure. In this context, a model is presented in which chemicals are evaluated based on inherent chemical properties and behaviorally-based usage characteristics over the chemical’s life cycle. These criteria are assessed and integrated within a decision analytic framework, facilitating rapid assessment and prioritization for future targeted testing and systems modeling. A case study outlines the prioritization process using 51 chemicals. The results show a preliminary relative ranking of chemicals based on exposure potential. The strength of this approach is the ability to integrate relevant statistical and mechanistic data with expert judgment, allowing for an initial tier assessment that can further inform targeted testing and risk management strategies.

## Introduction

Manufactured chemicals are widely used in products such as cosmetics, plastics, and electronics, and have applications in almost all industrial processes in sectors including energy, agriculture, and pharmaceuticals [1]. Increasing dependence on manufactured chemicals has not, however, been matched by an adequate increase in our understanding of the risks these may pose to the environment and human health [2]. Many chemicals in U.S. commerce today have unknown environmental fates and poorly understood potential for human exposure, including some of the most ubiquitous commercial chemicals, such as surfactants, fragrances, cleaning agents and pesticides [3], [4]. In this context, exposure is the contact of a stressor (i.e., a chemical agent) with a receptor (i.e., a human or a human population) for a specific duration of time [5]. Because of the lack of resources and sufficient scientific information on toxicity [6] and exposure [3] for the assessment of all chemicals, efforts are typically, and rationally, devoted to assessing those chemicals believed to pose the greatest potential risks based on production volume and chemical properties.

Within the domain of human health risk assessment, toxicity is an indication and measurement of the severity of adverse health effects a chemical causes in relation to an exposure level (dose). We broadly define exposure to be the contact of a stressor with a receptor for a specific duration of time [5]. The stressors of interest are chemical agents that can potentially lead to an adverse impact and the receptors of interest are individuals or population of individuals. Exposure is complex and dynamic in nature due to its spatial and temporal characteristics. For this reason, exposure-based prioritization efforts focus on relative exposure potential as a means to evaluate and rank chemicals. While prioritization is in of itself a risk management strategy, other risk management decisions may follow to include the allocation of scarce resources to complete future risk assessments, collection of additional data or testing, and/or (bio) monitoring. Therefore, the resolution and precision of the data incorporated in these efforts may vary according to the overall objective of the prioritization.

The U.S. EPA Office of Chemical Safety and Pollution Prevention recently performed a chemical prioritization exercise to identify 83 “TSCA Work Plan Chemicals” [7] as candidates for risk assessment during the next few years. Broad stakeholder input was used to identify prioritization and screening criteria and data sources. Chemicals were evaluated based on their combined hazard, exposure potential, and persistence and bioaccumulation characteristics using a two-step process. In the first step, a set of data sources was used to identify 1,235 chemicals meeting one or more criteria suggesting concern, namely: known reproductive or developmental effects; persistent, bioaccumulative, and toxic (PBT) properties; known carcinogenicity; and presence in children’s products. Excluding those chemicals not regulated under TSCA and those with physical and chemical characteristics that do not generally present significant health hazards narrowed the number of chemicals down to 345 candidates. In the second step, a numerical algorithm was used to score each chemical based on three characteristics: hazard, exposure, and potential for persistence or bioaccumulation. Candidate chemicals that ranked highest on the basis of their total score were identified as work plan chemicals; those that could not be scored because of an absence of exposure or hazard data were identified as candidates for information gathering.

Using the methodology described above, EPA has been able to identify a priority set of chemicals for near-term assessment based on criteria widely accepted as warranting concern. The scoring algorithm is transparent and the data sources are well documented. Focusing on chemicals with documented evidence of concern (i.e. “data-rich”) is reasonable in light of limited prototypes for *post hoc* screening and the paucity of available resources. However, this approach may not adequately address the need to make decisions about the thousands of chemicals in commerce and the hundreds of new chemicals introduced each year for which there is *little or n*o information [1], [3].

To support the development of novel rapid approaches for evaluating potential exposure of both existing and emerging chemicals, the EPA has initiated the ExpoCast research program [8]. This program is keenly interested in characterizing exposures across the chemical life cycle –manufacturing, transportation, product formulation, consumer product usage and finally disposal. EPA seeks to build on current chemical exposure models and knowledge to generate robust new protocols that better support chemical evaluation, risk assessment and risk management. Recent activities under this program have evaluated utility of available approaches for the purpose of rapidly prioritizing large numbers of chemicals on the basis of exposure [9], [10].

A number of exposure models were recently comparatively evaluated through the EPA Expocast model challenge, where a set of approximately 50 data-rich chemicals of different classes were ranked by several different approaches [10]. The chemicals were chosen to include high interest chemicals with a range of properties. Each modeling approach was capable of analyzing a different number of chemicals from the full set because of varying input requirements. Key findings of the comparative analysis among the prioritization schemes indicated significant differences in chemical ranking as a result of several factors: (1) which processes the model described across the source to effects continuum [11]; (2) the exposure metric or surrogate metric used for prioritization and which statistic (i.e., median, upper bound or lower bound estimate); (3) whether the model inputs included actual, modeled or unit emissions; (4) which exposure pathways were considered (i.e., from aggregated sources or through a dominant pathway); and (5) which type of exposure scenarios were considered (i.e., direct or indirect, diffuse source or concentrated source, etc.) [10]. Only mechanistic models characterizing exposure associated with environmental sources could rapidly evaluate and rank potential exposure for the majority of chemicals. To a great extent, this was due to both the minimum data requirements and the availability of predictive tools (i.e., QSARs) to generate model inputs that could be used to describe fate and transport under steady state and equilibrium conditions. Of the other models evaluated in the EPA Expocast model challenge, those designed for evaluation of chemicals in specific exposure scenarios lacked data for chemical and scenario specific input parameters and were thereby inhibited in their ability to produce ordinal rankings for the 55 chemicals.

Arguably, one of the major limitations of the models evaluated, and perhaps one of the larger knowledge gaps in exposure-based chemical prioritization itself, involves complex social behaviors that determine how humans come in contact with manufactured chemicals, particularly those emanating from near field sources (e.g., residential and consumer products). Thus there is a pressing need for enhancing current approaches with tools and techniques developed for understanding human behaviors, such as human factors engineering and marketing research, to better define scenarios describing how products are used. Accurate use scenarios among population groups of interest are necessary to properly characterize the consumer use component of a chemical’s life cycle.

Decision support tools borne out of the social sciences may also have a place in chemical prioritization. Multi-criteria Decision Analysis (MCDA), a rule-based method of classification for priority setting, is both a set of techniques and an approach for ranking alternatives [12], [13]. MCDA is a promising approach for exposure-based prioritization because it is transparent and understandable, yet complex and rigorous enough to include scenario-based reasoning, stochastic processes and value of information analysis. Moreover, it is amenable to sparse data [14], [15], [16], [17]. These characteristics complement some of the limitations of currently available statistical, mechanistic, or logic models, which provide useful frameworks for gathering relevant data but lack the social and policy context for risk-informed decision making. MCDA can merge a variety of types of exposure metrics from descriptions of physical chemical properties to the socioeconomic measures which characterize human activity, chemical use and contact to ultimately inform screening level risk estimates. Permitting structured integration of different types of information, MCDA methods provide a means for combining quantitative chemical property, production and use data with expert judgments and stakeholder preferences. MCDA assessment criteria can be adaptively weighted and modified in real time to evaluate both data-rich and data-limited chemicals.

Use of MCDA methods to support prioritization decision making under high uncertainty has been demonstrated many times including hazard identification and assessment. Risk management alternatives of industrial hazards or industrial consequences were relatively ranked using an MCDA approach by Paralikas and Lygeros [18]. The method recognizes that a single factor could not be used to define flammability and that different methods, tools, codes and legislation use varying sets of fire hazard properties as an example. Using the MCDA framework, the different decision criteria were successfully integrated using fuzzy logic to deal with linguistic variables and uncertainties allowing broad application for chemical hazard ranking decisions. In another example, life cycle assessment (LCA) was incorporated within a decision framework to prioritize future research and evaluate sensitivities to missing information in an assessment of processes for synthesizing single walled carbon nanotubes [14]. Engineered nanomaterials present uncertainties similar to chemicals in consumer products in terms of unknown environmental and human health across all life stages from formulation to disposal.

This paper demonstrates how analytical tools, such as LCA and MCDA, can offer a versatile and transparent approach to exposure-based prioritization utilizing results from several approaches evaluated in the EPA ExpoCast model challenge. The purpose of prioritization within this context is to focus resources on further evaluation of safety for chemicals with high potential for exposure and risk. A combination of exposure assessment model output with qualitative exposure criteria within such a decision framework has been recommended in the exposure-based waiving protocol within Europe’s REACH Regulation [19] which shares some similar goals for human and environmental health protection.

## Materials and Methods

**Figure 1:**
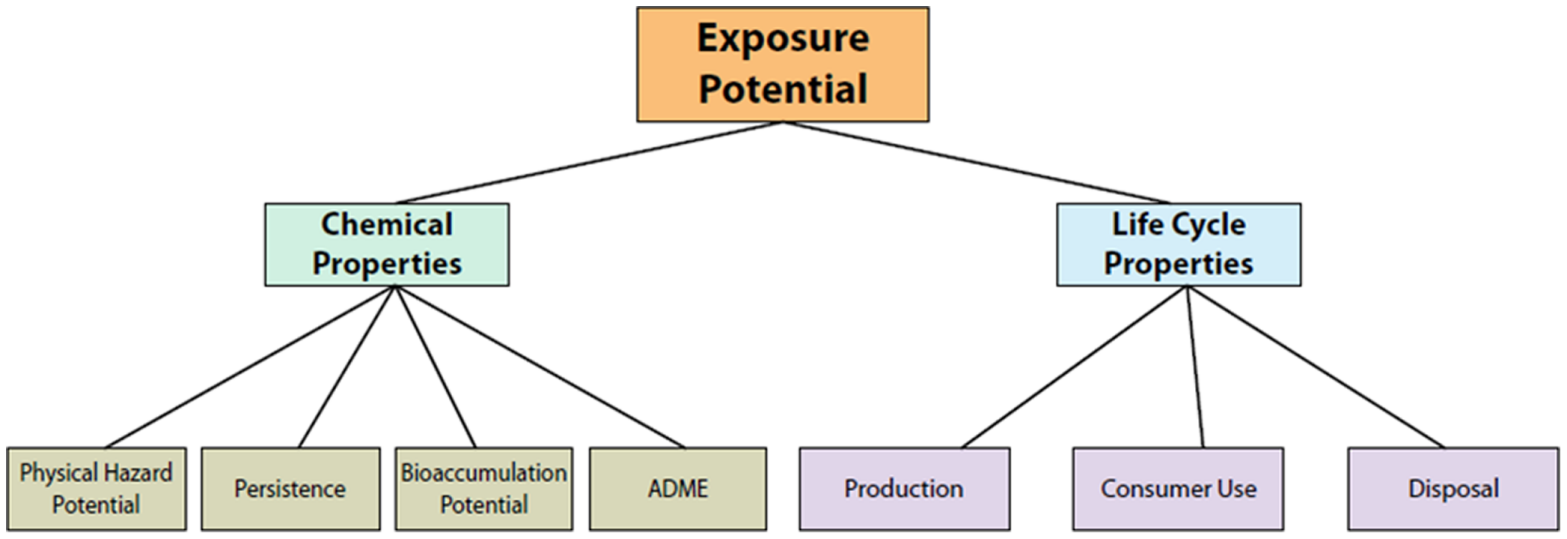
MCDA Framework for Exposure-Based Chemical Prioritization.

We propose a decision analytic approach for exposure-based chemical prioritization to address the need for novel, rapid exposure potential screening protocols. In this approach, we build on current research and existing models by evaluating relevant chemical exposure criteria within a larger MCDA framework. We employ a two-part prioritization model that incorporates both properties of the chemical itself and properties of the chemical’s life cycle (**Figure 1**).

The chemical property and life cycle property assessments are structured to analyze exposure-related information associated with specific chemical properties and distinct life cycle phases, respectively. Relevant chemical and life cycle properties are grouped into several criteria based upon the means by which each property contributes to the chemical’s overall exposure potential (e.g., properties associated with a chemical’s ability to bioaccumulate vs. those associated with its ability to be metabolized by the human body). Chemical and life cycle properties in each criterion are then further divided into various sub-criteria. The numerical values associated with these properties for a given chemical serve as inputs to the model. Input data can be obtained from a number of different sources, including existing databases, current literature and expert judgment. The criteria within this decision model were selected by reviewing those used in the models submitted to the ExpoCast model challenge, [10] and then structured into a hierarchical framework based on discussions with exposure science experts.

Within each sub-criterion, the constituent chemical or life cycle property is evaluated to determine its contribution to overall exposure potential. Input values for individual properties are compared against established numerical thresholds, which define distinct levels of risk that span the range of possible values for the given sub-criterion. Thresholds are used to score property values based on the indicated level of risk (e.g., a compound with a longer half-life may have higher potential for exposure than a compound with a shorter half-life, all other things being equal).

Following an MCDA approach, sub-criterion scores are then combined according to explicit decision rules to derive scores for their higher-level criterion. Chemical property and life cycle phase criterion scores are then combined to produce a Chemical Properties Exposure Score (CPES) and a Life Cycle Exposure Score (LCES) for each chemical. These scores reflect relative estimates of chemical exposure potential as indicated by available chemical property and life cycle property data, respectively. Exposure scores may then be integrated to derive aggregate measures of exposure potential, which can be used to compare and prioritize chemicals on a relative basis, or can remain separate and be plotted on a risk matrix for a more qualitative assessment.

Chemical property and life cycle phase criteria can be weighted within each assessment to reflect their relevance to the user’s management objectives. Weights may indicate a specific focus of the assessment or reflect expert judgment of a criterion’s predictive reliability or relative importance. Criterion weights can be adjusted to refine the scope of a particular assessment to a particular class of chemicals (e.g., pesticides), a particular exposure scenario (e.g., occupational exposure), or a particular exposure target (e.g., environmental contamination). When eliciting subjective weights, it is important to utilize best practices to avoid potential biases and inconsistencies [20], [21]. Numerous elicitation techniques exist, including rank-based methods and swing-weight methods [13], [21], [22].

### Chemical Properties Assessment

As seen in **Figure 1**, the Chemical Properties Assessment considers four main criteria to estimate potential risk for human exposure: bioaccumulation potential, persistence, ADME (**A**bsorption, **D**istribution, **M**etabolism, and **E**limination), and physical hazard potential. Each criterion constitutes a unique set of sub-criteria, which define the distinct chemical property data points that serve as inputs to the assessment. Observed chemical properties used to estimate exposure potential are defined by the specific sub-criteria under each of the four main criteria. Using thresholds established for each sub-criterion, individual data points are evaluated and assigned scores representing the potential for exposure indicated by the observed chemical property. Once these initial scores have been calculated, the highest within each set of sub-criteria is assigned as that criterion’s exposure score.

When certain chemical-specific data are unavailable, as is often the case in this context, it may not be possible to assign scores to each sub-criterion. By defining each criterion’s exposure score as the highest of its associated sub-criteria scores, we account for this possibility. By employing this approach, criterion scores can be assigned even in the presence of sparse data.

Each chemical’s bioaccumulation, persistence, ADME, and physical hazard scores are combined with their associated weights. Weighted criteria exposure scores are then summed to produce initial chemical property exposure score for each chemical. Once this has been done for the set of chemicals being assessed, the initial chemical property exposure scores are normalized from 0 to 1 to produce relative rankings.

#### Bioaccumulation

Bioaccumulation is a process in which a chemical substance is absorbed by an organism via all routes of exposure in the natural environment, for example through dietary and ambient environmental sources, and increases in concentration over time [23]. Using three bioaccumulation-related sub-criteria, we evaluate surrogate chemical properties in order to predict the compound’s ability to bioaccumulate.

#### Bioconcentration factor (BCF)

A compound’s BCF is a dimensionless number representing the relative concentration of the compound in organic tissues. In general, chemicals with relatively higher BCFs have greater potential for exposure, and thus are more likely to adversely impact human health and the environment. In this model, four distinct numerical thresholds were used to evaluate chemical BCF data. These thresholds are shown in **Table 1**, and were used to assign each chemical a BCF sub-criteria score from 1–4 based on the indicated level of bioaccumulation potential. Thresholds are based on previously published values employed by existing exposure assessment models: the EPA Design for the Environment Program [24], and the Clean Production Action’s Green Screen for Safer Chemicals Initiative [25]. To address minor numerical discrepancies, the more conservative thresholds were chosen when values differed between models.

**Table 1:** Thresholds for Bioaccumulation Potential and Environmental Persistence.

Score	1 (Low)	2 (Moderate)	3 (High)	4 (Very High)
**Bioaccumulation**				
BCF	<100	>100 to 1000	>1000 to 5000	>5000
Log Kow	<2	>2 to 3	>3 to 5	>5
**Persistence**				
Half Life in Water	<168 days	>168 to 960 days	>960 to 1440 days	>1440 days
Half Life in Soil	<384 days	>384 to 1440 days	>1440 to 4320 days	>4320 days
Half Life in Sediment	<384 days	>384 to 1440 days	>1440 to 4320 days	>4320 days
Half Life in Air	<2	n/a	> = 2	n/a

#### Log kow

A compound’s K_ow_, or octanol-water partition coefficient, describes its ability to transition between water and carbon-based media. Chemical compounds with relatively higher log K_ow_ are capable of greater movement within the environment; they are thus more adaptive and have higher potential for human exposure and absorption. In this model, four distinct numerical thresholds were used to evaluate chemical K_ow_ data. These thresholds are shown in **Table 1**, and were used to assign each chemical a log K_ow_ sub-criteria score from 1–4 based on the indicated level of bioaccumulation potential. Thresholds are based on previously published values employed by existing exposure assessment models: the EPA Design for the Environment Program [24], and the Clean Production Action’s Green Screen for Safer Chemicals Initiative [25], with the more conservative threshold chosen when values differed between models.

#### Molecular weight

Previous studies have identified a significant correlation between a compound’s molecular weight and its ability to bioaccumulate [26], [27]. Results from these studies support the general conclusion that heavy molecules do not easily bioaccumulate, as their size hinders passage through lipid membranes. Lower weight chemicals thus possess a relatively greater potential for human exposure. These and similar findings have been used to inform chemical testing policy and legislation such as the OECD Chemical Substance Control Law (CSCL) in Japan [28] and the EPA Toxic Substances Control Act (TSCA) in the United States [29].

A single cut-off threshold is employed by our model to evaluate molecular weight data. Molecules 1000 amu or greater are given a bioaccumulation criteria score of 1, regardless of their other sub-criteria scores within the bioaccumulation category (BCF & log Kow). The 1000 amu cut-off follows TSCA premanufacture notification policy [29], and is based on current understanding that molecular weights in this range are generally better indicators of chemical bioaccumulation potential than other surrogate properties [26].

#### Persistence

Persistence corresponds to the length of time a chemical can exist in the environment before degrading or being transformed by natural processes [23]. Persistent chemicals are more likely to come into contact with humans compared to chemicals that degrade quickly in the environment. We consider the half-life in water, soil, sediment, and air for each chemical as surrogate indicators of persistence for the purpose of evaluating exposure potential.

The numerical thresholds used for evaluating chemical half-life data are shown below in **Table 1**. Thresholds were used to assign each chemical four distinct half-life sub-criteria scores from 1–4 based on the level of persistence indicated by each of the four half-lives (in water, soil, sediment, and air). Threshold values for water, soil, and sediment are based on previously published values employed by existing exposure assessment models: the EPA Design for the Environment Program [24], and the Clean Production Action’s Green Screen for Safer Chemicals Initiative [25], using the more conservative thresholds. The threshold value for air follows science-based guidance for evaluating chemical long-range transport potential and overall persistence [30]. Chemicals with half-lives in air that are less than two days are assigned an associated sub-criteria score of 1 (“Low”), while those with half-lives in air greater than or equal to two days are assigned an score of 3 (“High”).

#### ADME

Properties that describe a chemical’s ability for absorption, distribution, metabolism, and excretion (ADME) are indicators of the potential for biologically relevant human exposure. Chemicals that can be easily absorbed by the body and that are resistive to metabolism or excretion pose a greater threat for extended exposure; therefore it is useful to focus on the entrance and exit of the chemicals within the context of the body. Though recent and current ADME-related research efforts have focused on establishing appropriate surrogate properties and developing predictive models, general consensus has not been reached regarding an accepted approach to ADME assessment for environmental chemicals [10]. Building on current research and existing models, a new ADME assessment protocol intended for screening-level exposure-based chemical prioritization was incorporated into the framework [10]. This method utilizes QikProp software Version 3.0 [31], a QSAR-based model to obtain surrogate chemical property values, which were then integrated to evaluate ADME properties along various sub-criteria briefly discussed below. All QikProp values are based on a 24-hour exposure period. Incidentally, QikProp is a three-dimensionally based structure method, so the SARs depend on the solvent accessible surface area. The properties calculated are dependent on the conformer adopted at the time of calculation and could be sensitive to molecular orientation. In addition, QikProp was designed exclusively to develop organic pharmaceutical compounds, so cannot be used for metals and inorganic compounds. Thus, if the analytics discussed herein are to be applied to metals and inorganic compounds, another QSAR system is needed.

#### Absorption

The chemical absorption assessment is based on two QikProp predictors which describe oral availability. The first descriptor represents a qualitative measure of oral absorption potential, and takes values of 1, 2, or 3 for low, medium, or high, respectively. The second descriptor represents a numerical probability of oral absorption on a 0 to 100% scale, with <25% and >80% designating low and high probability, respectively. These values were combined to derive an absorption score (1–3) for each chemical.

Distribution/Excretion: Distribution and excretion-related properties were combined into a single assessment. QikProp predicted octanol/water partition coefficients, serving as surrogates for half-life within the human body, were categorized into bins using subjective thresholds to derive a distribution/excretion score (1–4) for each chemical.

#### Metabolism

The assessment of metabolism was derived from the QikProp descriptor representing the number of expected possible metabolites for each chemical over a 24-hour period in the human body. These values were categorized based on the predicted half-life of each chemical in order to represent metabolism via natural degradation in the body. These values were combined to generate average metabolism scores (1–4) for each chemical.

#### Physical hazard potential

Highly flammable and reactive chemicals pose human and environmental threats that may not be considered in standard exposure or toxicity-based assessments. Though the properties that determine a given chemical’s flammability and reactivity may be distinct from those that determine its environmental fate and transport, the threat of physical hazard is nonetheless directly related to the likelihood of exposure. The risk of physical hazards (e.g., combustion) is thus an exposure-related risk, and we assess each chemical’s hazard-related properties in order to anticipate threats that may not be considered in other exposure or toxicity-based screenings. In accordance with existing National Fire Protection Association (NFPA) standards and classifications [32], flammability and reactivity were assigned scores of (1–4) using established NFPA thresholds.

### Chemical Life Cycle Properties Assessment

Similarly to the assessment of chemical properties, we estimate potential for human exposure by assessing three main life cycle phases of manufactured chemicals: production, consumer use, and disposal. Each phase constitutes a unique subset of exposure-related criteria, which define the distinct life cycle characteristics that serve as inputs to the assessment.

The different criteria associated with each of the three life cycle phases designate the individual life cycle properties that will serve as indicators of a chemical’s exposure potential during the relevant phase. All life cycle criteria are evaluated quantitatively, with higher values indicating higher potential for exposure. Instead of establishing thresholds for each sub-criteria as in the assessment of chemical properties, raw values are used but then normalized across the set of chemicals for each individual sub-criteria. This provides bounds for the range of values and assists in making comparative assessments.

Criteria scores are then calculated by summing the sub-criteria scores. Again, these scores are normalized across the set of chemicals to account for criteria containing more sub-criteria than others, and then multiplied by their weights to produce an initial Life Cycle Properties Exposure Score (LCES). Once initial LCESs have been calculated for all chemicals, we derive final LCESs by normalizing initial scores to the highest and lowest observed scores across all chemicals.

#### Production

Number of Potential Exposure Sources*:* Each chemical is evaluated to determine the possibility for human exposure during processes associated with production of the chemical. We consider one potential source (*occupational microenvironments*) defined as any workplace environment in which a release might occur during chemical manufacture and/or processing. Each chemical is assigned a score of either 0 or 1 based on whether the compound presents risk of exposure during production.

#### Projected average annual number of production sites

A chemical’s exposure risk is increased if it is produced in many locations. Ubiquity classifications for each chemical were used to estimate the amount of chemical production sites [10]. Higher scores indicate increased potential for human exposure during chemical production: very widespread (5), widespread (4), moderate (3), localized (2), low (1).

#### Regional geometric mean production quantity (MQR)

In addition to how widespread production is, estimates are made of the quantity produced. This is estimated using the Regional Geometric Mean Production Quantity (MQ_R_), measured in units of kilotons per year. This is an estimated quantity, but production quantities could also be provided by industry.

### Consumer Use

The assessment evaluates several sub-criteria relevant to the consumer use phase in the life cycle of manufactured chemicals. Based on the intended uses of each chemical, primary consumer class is defined as either strictly industrial, or industrial *and* individual. Chemicals used during industrial processes (e.g., monomers, solvents) and chemicals otherwise noted to have primarily industrial consumers were defined to have a strictly industrial consumer class. Chemicals used in agriculture (e.g., pesticides, insecticides, herbicides) or as food/cosmetic additives (e.g., preservatives, anti-microbials) were defined to have both industrial and individual consumers. Chemicals directly incorporated into consumer products during their production (e.g., plastics, coatings, fabrics, flame retardants) are also defined to have both industrial and individual consumers.

#### Number of potential exposure sources

Each chemical was evaluated to determine the possibility for human exposure during processes associated with both industrial and individual consumer uses of the chemical. Ten distinct potential sources associated with consumer exposure were considered (i.e., outdoor air, water, soil, biota, indoor air/dust, in-vehicle air, object contact, tap water, other water, food/beverages) by assigning each chemical a score from 0–10 based on possibility for exposure via each unique source during consumer use of the compound.

#### Projected average annual number of individual consumers

Chemicals defined as having industrial *and* individual consumer classes were assessed to determine their potential for exposure to individual consumers in non-industrial settings. Chemical ubiquity classifications were used to represent the relative size of each chemical’s average, annual, individual consumer base. Chemicals defined as having strictly industrial consumer classes were assigned individual consumer scores of 0. Remaining chemicals were assigned scores from 1–5 based on their ubiquity, with higher scores indicating increased potential for individual consumer exposure during non-industrial use: very widespread (5), widespread (4), moderate (3), localized (2), low (1).

#### Projected average annual number of industrial consumers

To assess chemicals’ potential for exposure to industrial consumers, we employ the ubiquity classification to estimate the average, annual size each chemical’s industrial consumer base. As none of the chemicals assessed were defined as having a strictly individual (non-industrial) consumer base, all chemicals were assigned scores from 1–5 based on their ubiquity classification, with higher scores indicating increased potential for industrial consumer exposure during use of the chemical: very widespread (5), widespread (4), moderate (3), localized (2), low (1).

#### Projected average annual quantity consumed per individual/industrial consumer

The average annual quantity of each chemical consumed per consumer was predicted using the relative size of the chemical’s total consumer base (including both individual and industrial consumers), and its MQ_R_. Relative measures of consumption quantity per consumer (Q) were calculated by dividing each chemical’s projected mean production volume by their total number of consumers, assuming chemicals with higher consumption quantities to have increased potential for consumer exposure. Projected annual quantities consumed per individual consumer were calculated using the same equation as that for industrial consumers:
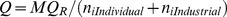
(1)where (n_iIndividual_+n_iIndustrial_) represents the chemical’s total consumer base, or the number of individual consumers plus the number of industrial consumers.

#### Susceptible populations

To determine if there was a heightened exposure risk to susceptible populations (in this case, children), particular processes associated with individual consumer use of the chemical were evaluated. Nine distinct potential sources associated with exposure to children were considered (Outdoor Air, Water, Soil, Indoor Air/Dust, In-Vehicle Air, Object Contact, Tap Water, Other Water, and Food/Beverages), and each chemical was assigned a score from 0–9 based on possibility for exposure via each unique source.

### Disposal

#### Number of potential exposure sources

Each chemical was evaluated to determine potential for human exposure resulting from disposal events. We consider four distinct disposal-related sources (Outdoor Air, Water, Soil, Biota), assigning each chemical a score from 0–4 based on potential for exposure via each unique source during and after disposal of the compound.

#### Projected average annual number of disposal events

Each chemical’s total number of consumers was estimated to determine an annual number of associated chemical disposal events. Assuming that each chemical’s industrial and individual consumers dispose of equal amounts of the compound, we define the projected number of disposal events as each chemical’s total number consumers, and assign scores of 1–10, with higher scores representing greater potential for disposal-related human exposure.

#### Projected average annual quantity disposed

To account for assumed variations in the actual quantities disposed during industrial and individual consumer disposal events, we assume that 0.1% of the net production volume of each chemical is disposed of in order to evaluate disposal-related exposure potential. Note that the use of this unit value assumes that no chemical- or product-specific data were available. With larger disposal quantities indicating higher potential for post-disposal chemical exposure, we calculate relative disposal quantities of each chemical (*Q_DISP_*) as:
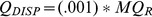
(2)


### Integrating Chemical Properties and Life Cycle Exposure Scores

Once assessments of chemical properties and life cycles have been performed on all chemicals, those chemicals lacking sufficient data to calculate either a chemical properties exposure score or life cycle exposure score are removed from the remainder of the prioritization. Though these chemical’s available scores may indicate significant threat of exposure, they are excluded from the integration process as their scores can skew final exposure potential relationships. The remaining chemicals are renormalized as:
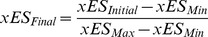
(3)where *xES* denotes the relevant exposure score (either chemical or life cycle). Next, the remaining chemicals’ exposure scores (chemical property and life cycle property) are summed to produce aggregate exposure scores. These scores represent cumulative measures of exposure potential based on each chemical’s distinct properties and characteristics of its projected life cycle. Aggregated exposure scores, which all lie in the range of 0–2, are used to numerically rank chemicals based on their potential for human exposure.

**Figure 2:**
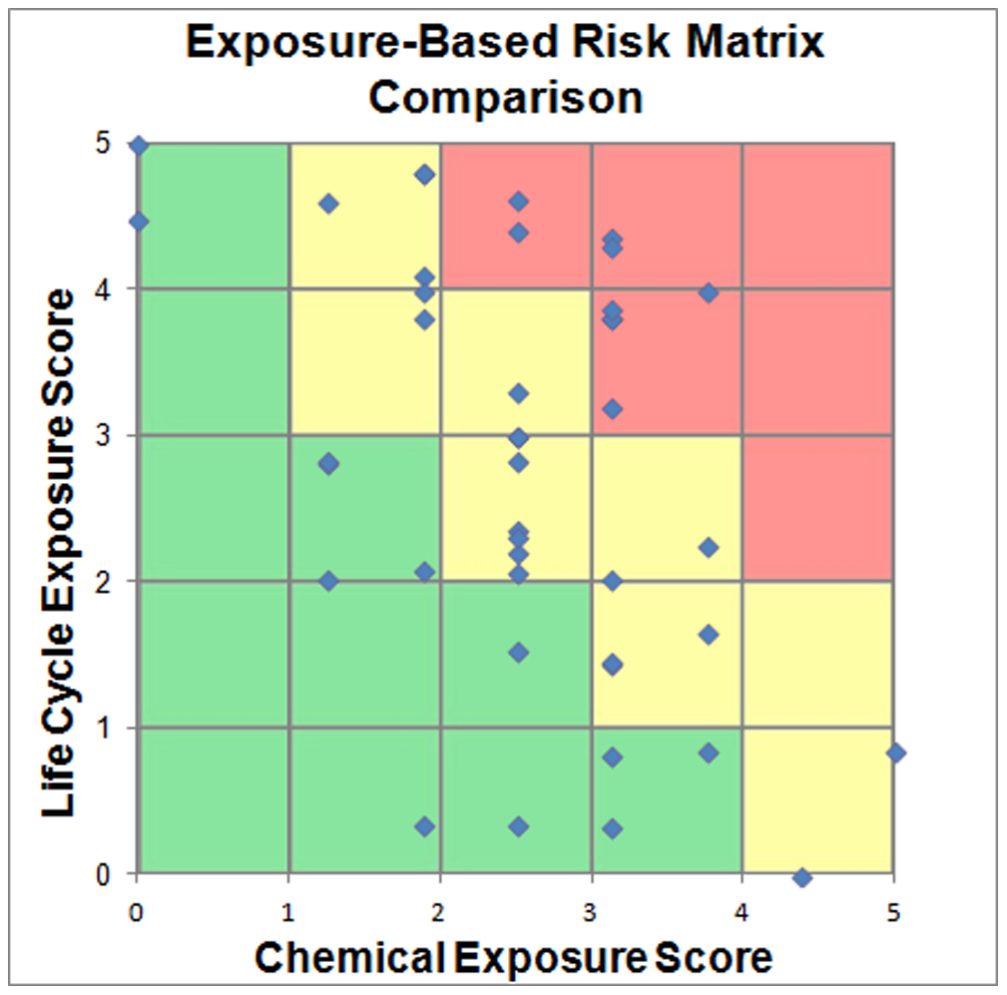
Example Chemical Exposure Potential Risk Matrix.

In addition to this quantitative integration, chemical property and life cycle scores can be visualized using a risk-reporting matrix (**Figure 2**) for a more qualitative assessment of aggregate chemical exposure potential.

In this method of integration, chemical property and life cycle exposure scores are converted from a scale of 0–1 to a scale of 0–5 by multiplying the initial score by a factor of five to place them within the 5×5 risk matrix, with each chemical’s position representing a qualitative, cumulative measure of exposure potential based on both chemical and life cycle properties. Qualitative exposure potential thresholds (red, yellow, or green) can be defined within the matrix to designate high, moderate, and low risk regions.

## Case Study

### Data Set

For the case study, a set of 51 chemicals was selected from those presented and evaluated in the model challenge (**Table 2**), representing a wide variety of chemical classifications (e.g., organics, metals, etc.). Sub-criteria scores for these chemicals were collected from numerous reports and online databases, and the sources for each sub-criterion are listed in **Table 3**. Case study data can be found in the online File S1.

**Table 2:** Case Study Chemicals.

Chemical	CAS #	Chemical	CAS #
Formaldehyde	50000	Malathion	121755
DDT	50293	Perchloroethylene	127184
Parathion	56382	1-methoxy-4-(2-propen-1-yl)-benzene	140670
gamma-Hexachlorocyclohexane	58899	decaBDE	1163195
Carbaryl	63252	Trifluralin	1582098
Methoxychlor	72435	PFOS	1763231
Vinyl Chloride	75014	Atrazine	1912249
1,1,2,2-tetrachloroethane	79345	Lead	7439921
Tetrabromobisphenol A	79947	Manganese	7439965
Bisphenol-A	80057	Cadmium	7440439
p-tert-Pentylphenol	80466	Butylhydroxyanisole	8003245
Diethyl phthalate	84662	Perchlorate (Mg salt)	10034818
Di-n-butylphthalate	84742	Tris (l,3-dichloro-2-propyl) phosphate	13674878
1,2,3 Trichlorobenzene	87616	Methyl mercury	22967926
Pentachlorophenol	87865	Phenol, (l,l-dimethylethyl)-4-rnethoxy	25013165
2,4,5-Trichlorophenoxy acetic acid	93765	Nonylphenol	25154523
2,4-D	94757	Hexabromocyclododecane (HBCD)	25637994
Ethylene thiourea	96457	8-2 fluorotelomer acid	27854315
Methylparaben	99763	Aroclor_1260	35065271
Styrene	100425	Aroclor_1254	38380017
n-Hexane	110543	Vinclozolin	50471448
Tris (2-chloroethyl) phosphate	115968	Permethrin	52645531
Aldicarb	116063	Penta BDE	60348609
DEHP, Di(2-ethylhexyl)phthalate	117817	C10–C13 Chloroalkanes	85535848
Hexachlorobenzene	118741	octaBDE	207122165
Ethylparaben	120478		

**Table 3:** Data Sources.

Criteria	Sub-Criteria	Data Sources
**Chemical Properties**		
ADME	Absorption (A)	QikProp software Version 3.0 [31]
ADME	Distribution/Excretion (D/E)	QikProp software Version 3.0 [31]
ADME	Metabolism (M)	QikProp software Version 3.0 [31]
Bioaccumulation	Bioconcentration Factor (BCF)	PBT Profiler [33]; Estimation Programs Interface Suite™ (EPI suite) [23]
Bioaccumulation	Log Kow	EPA Exposure-Based Prioritization Challenge [34]
Bioaccumulation	Molecular Weight	EPA Exposure-Based Prioritization Challenge [34]
Persistence	Half Life in Air	EPA Exposure-Based Prioritization Challenge [34]; Mitchell, et al. [10]
Persistence	Half Life in Water	EPA Exposure-Based Prioritization Challenge [34]; Mitchell, et al. [10]
Persistence	Half Life in Soil	EPA Exposure-Based Prioritization Challenge [34]; Mitchell, et al. [10]
Persistence	Half Life in Sediment	EPA Exposure-Based Prioritization Challenge [34]; Mitchell, et al. [10]
Physical Hazard	Flash Point (Flammability)	Material data safety sheets
Physical Hazard	Explosivity (Reactivity)	Material data safety sheets
**Life Cycle Properties**		
Production	Number of Potential Exposure Sources	EPA Exposure-Based Prioritization Challenge [34]
Production	Projected Avg. Annual Number ofProduction Sites	EPA Exposure-Based Prioritization Challenge [34]
Production	Regional Geometric Mean Production Quantity [MQR]	EPA Exposure-Based Prioritization Challenge [34]
Consumer Use	Number of Potential Exposure Sources	EPA Exposure-Based Prioritization Challenge [34]
Consumer Use	Projected Avg. Annual Number of IndividualConsumers	EPA Exposure-Based Prioritization Challenge [34]
Consumer Use	Projected Avg. Annual Number of IndustrialConsumers	EPA Exposure-Based Prioritization Challenge [34]
Consumer Use	Projected Avg. Annual Quantity ConsumedPer Individual Consumer	EPA Exposure-Based Prioritization Challenge [34]
Consumer Use	Projected Avg. Annual Quantity Consumed PerIndustrial Consumer	EPA Exposure-Based Prioritization Challenge [34]
Consumer Use	Susceptible Populations: Number of PotentialExposure Sources to Children	EPA Exposure-Based Prioritization Challenge [34]
Disposal	Number of Potential Exposure Sources	EPA Exposure-Based Prioritization Challenge [34]
Disposal	Projected Avg. Annual # of Disposal Events	EPA Exposure-Based Prioritization Challenge [34]
Disposal	Projected Avg. Annual Quantity Disposed	EPA Exposure-Based Prioritization Challenge [34]

### Prioritization

First, the data for each chemical was compiled. It was found that some chemicals were difficult to assess due to a lack of readily available data. If a chemical did not have any sub-criteria scores for at least one of its criteria, that chemical was removed from the analysis process as having too little data for analysis. Nine of the 51 chemicals (largely metals) were removed for this reason.

**Figure 3:**
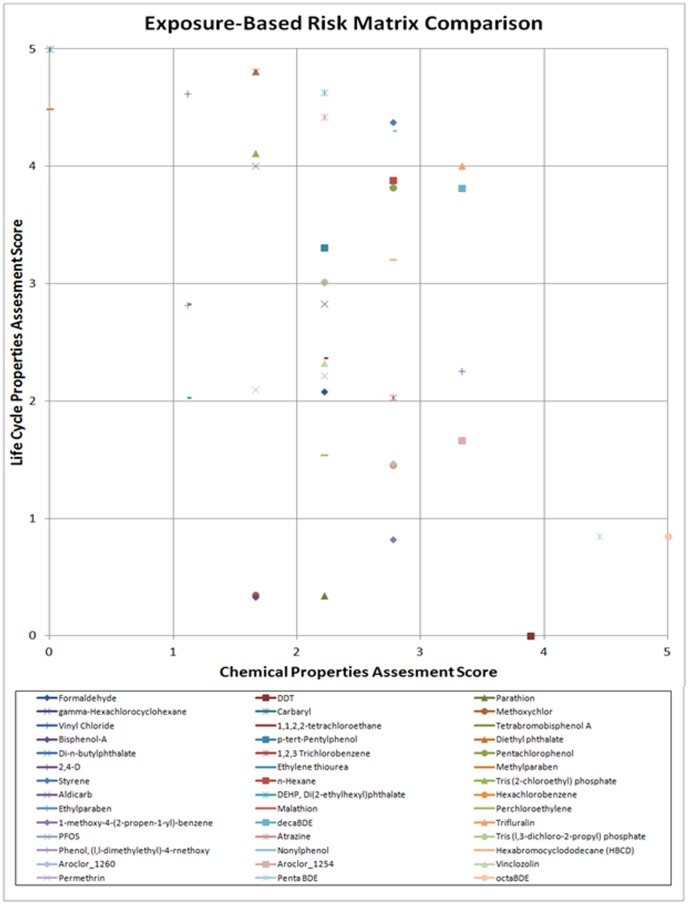
Risk Matrix Comparison of Exposure Potential with Even Weighting.

Following the MCDA approach outlined above, each of the remaining test chemicals was assessed. Scores for each criterion were weighted by allocating equal weights (i.e., bioaccumulation, persistence, ADME, and physical hazards each weighted 25%; production, consumer use, disposal each weighted 33.33%). The final prioritization under this weighting distribution is shown in **Table 4**. The risk matrix comparison under this weighting distribution is shown in **Figure 3**.

**Table 4:** Exposure Rankings with Even Weighting.

Rank	Chemical Name	CAS #	Chemical Property Score	Life Cycle Score	Aggregate Exposure Score
**1**	Trifluralin^3^	1582098	0.67	0.80	**1.47**
**2**	Styrene^2^	100425	0.56	0.87	**1.43**
**3**	decaBDE^4^	1163195	0.67	0.76	**1.43**
**4**	Nonylphenol^4^	25154523	0.56	0.86	**1.42**
**5**	DEHP, Di(2-ethylhexyl)phthalate^2^	117817	0.44	0.92	**1.37**
**6**	n-Hexane^4^	110543	0.56	0.78	**1.33**
**7**	Atrazine^3^	1912249	0.44	0.88	**1.33**
**8**	Tetrabromobisphenol A^2^	79947	0.56	0.76	**1.32**
**9**	Pentachlorophenol^3^	87865	0.56	0.76	**1.32**
**10**	Di-n-butylphthalate^2^	84742	0.33	0.96	**1.29**
**11**	Diethyl phthalate^2^	84662	0.33	0.96	**1.29**
**12**	Hexabromocyclododecane (HBCD)^2^	25637994	0.56	0.64	**1.20**
**13**	octaBDE^2^	207122165	1.00	0.17	**1.17**
**14**	Tris (2-chloroethyl) phosphate^2^	115968	0.33	0.82	**1.15**
**15**	2,4-D^3^	94757	0.22	0.92	**1.15**
**16**	Aldicarb^3^	116063	0.33	0.80	**1.13**
**17**	Vinyl Chloride^1^	75014	0.67	0.45	**1.12**
**18**	p-tert-Pentylphenol^3^	80466	0.44	0.66	**1.11**
**19**	Penta BDE^4^	60348609	0.89	0.17	**1.06**
**20**	Tris (l,3-dichloro-2-propyl) phosphate^2^	13674878	0.44	0.60	**1.05**
**21**	Phenol, (l,l-dimethylethyl)-4-rnethoxy^5^	25013165	0.44	0.60	**1.05**
**22**	gamma-Hexachlorocyclohexane^3^	58899	0.44	0.57	**1.01**
**23**	Carbaryl^3^	63252	0.00	1.00	**1.00**
**24**	Aroclor_1254^1^	38380017	0.67	0.33	**1.00**
**25**	1,2,3 Trichlorobenzene^3,4^	87616	0.56	0.41	**0.96**
**26**	1,1,2,2-tetrachloroethane^1^	79345	0.44	0.47	**0.92**
**27**	Vinclozolin^3^	50471448	0.44	0.46	**0.91**
**28**	Methylparaben^5^	99763	0.00	0.90	**0.90**
**29**	PFOS^4^	1763231	0.44	0.44	**0.89**
**30**	Formaldehyde^1,4^	50000	0.44	0.42	**0.86**
**31**	Aroclor_1260^1^	35065271	0.56	0.29	**0.85**
**32**	Hexachlorobenzene^3,4^	118741	0.56	0.29	**0.85**
**33**	Malathion^3^	121755	0.22	0.57	**0.79**
**34**	Ethylparaben^5^	120478	0.22	0.56	**0.79**
**35**	DDT^3^	50293	0.78	0.00	**0.78**
**36**	Perchloroethylene^1^	127184	0.44	0.31	**0.75**
**37**	Permethrin^3^	52645531	0.33	0.42	**0.75**
**38**	1-methoxy-4-(2-propen-1-yl)-benzene^5^	140670	0.56	0.16	**0.72**
**39**	Ethylene thiourea^3,4^	96457	0.22	0.41	**0.63**
**40**	Parathion^3^	56382	0.44	0.07	**0.51**
**41**	Methoxychlor^3^	72435	0.33	0.07	**0.40**
**42**	Bisphenol-A^2^	80057	0.33	0.07	**0.40**
	2,4,5-Trichlorophenoxy acetic acid^3^	93765	**n/a**	**n/a**	Insufficient Data
	Lead^4^	7439921	**n/a**	**n/a**	Insufficient Data
	Manganese^5^	7439965	**n/a**	**n/a**	Insufficient Data
	Cadmium^4^	7440439	**n/a**	**n/a**	Insufficient Data
	Butylhydroxyanisole^5^	8003245	**n/a**	**n/a**	Insufficient Data
	Perchlorate (Mg salt)^1^	10034818	**n/a**	**n/a**	Insufficient Data
	Methyl mercury^1^	22967926	**n/a**	**n/a**	Insufficient Data
	8-2 fluorotelomer acid^4^	27854315	**n/a**	**n/a**	Insufficient Data
	C10–C13 Chloroalkanes^4^	85535848	**n/a**	**n/a**	Insufficient Data

Key:

1 Industrial/occupational additives and byproducts.

2 Plastics.

3 Pesticides and herbicides.

4 Additives in commercial products.

5 Additives in food and commercial products.

## Discussion

As stated above, one of the major limitations of currently available exposure models involves the inability to fully characterize the influence of complex social behaviors on resulting exposures or contact between humans and manufactured chemical across all life stages of the chemical. This is especially true for chemicals used in residential and consumer products, those arising from near field sources. A multi-criteria decision model was developed to combine typical physiochemical screening level data with measures to characterize human activities. As a proof of concept to show the utility of this approach, a case study was conducted on a small set of chemicals that were also analyzed using higher tiered statistical and mechanistic exposure models in a model challenge [10]. The models used in the model challenge considered different types of exposure scenarios including indirect exposures from diffuse environmental sources and direct, concentrated exposures from micro-environmental sources (i.e. from a personal care product or within a residence), though the latter had significant limitations in terms of necessary data to produce exposure estimates. Ranking results were obtained by three models and the comparative analysis is reported elsewhere [10]. Some agreement between ranking results was observed, but in general these models produced widely incongruous results across a number of different domains of information. Interestingly, some of the results using the MCDA model developed herein coincide with results from these more complex models. The majority of the chemicals (13 of 14) ranked in the top one-third of the list in **Table 4** (Rank 1–14), are also ranked in the top one-third of one of the models evaluated in the challenge. In general this agreement is with a “far-field” indirect diffuse source model which does not incorporate human activity at the micro-environmental level. Nonylphenol was the exception as it was ranked low by all other mechanistic models. Similarly, the bottom third of the ranked list in **Table 4** (Rank 28–42) shows high agreement with results from a model from the challenge. One model used characterized both far-field and near-field exposures and the other two were far-field models.

Because this analysis was conducted as a proof of concept, an exhaustive search for quality data and subsequent data validation was not conducted independently of the model challenge. However, the absence of the mechanistic relationships involved in the exposure models as well as the equal weighting scheme used in our example would lead to the assumption that the input drivers of the challenge models would be different than the input drivers of MCDA model. To fully explore this assumption and the utility of this methodology for larger scale research prioritization or policy guidance, the results of the case study underscore the need for quality data inputs. Only nine of the chemicals had to be excluded. These chemicals have properties that exclude them from the domain of applicability of the analytics, e.g. models, QSAR type, and other tools. As mentioned, metals and inorganic compounds are not characterized by the ADME models used in this study.

For the majority of compounds that fall within the domain of applicability, the MCDA approach is useful. As shown in **Table 4**, the majority of the chemicals used in plastics appear in the top half of the ranked list denoting highest exposure potential by highest aggregated exposure score. Plastics are broadly related to exposures that occur in all locations across the life-cycle of the chemicals. The chemicals in the bottom half of the ranked list (lower exposure potential) fit into a number of other of categories, but 11 of 21 are or were used as pesticides/herbicides, agriculturally, in homes or in public and commercial areas. The two pesticides/herbicides, Parathion and Methoxychlor, are ranked relatively low on the list in **Table 4**. Both chemicals were exclusively used in agriculture only, but have been previously banned or restricted by the EPA and do not have other uses like 1,2,3-trichlorobenzene, ethylene thiourea, and hexachlorobenzene which were also used exclusively in agriculture but are now used as a nonfood commercial additives. The remaining chemical in the agricultural only category is aldicarb. Aldicarb was restricted more recently in 2010 and will not be completely phased out until 2018, so exposure potential may be higher than the others in this category.

It should be noted that the nature of this analysis is to score chemicals in a comparative and relative manner, as opposed to assigning an absolute measure of exposure risk, which would not be practical or appropriate for a screening tool such as this. The relative assessment of chemical exposure potential is therefore dependent upon the set or sub-set of chemicals under consideration, and must be considered when designing the analysis and interpreting the results.

If a risk matrix is used for interpretation or communication of exposure potential results, it is important to note that a chemical with a high chemical property score and low life cycle property score (or vice versa) may be displayed has having a low exposure risk. When the risk matrix is used for score integration, however, these chemicals will appear on the boundaries of the matrix and can easily be identified as outliers that may warrant further assessment. Figure 3 shows the results of the case study on such a risk matrix. The risk matrix approach can be used to graphically visualize qualitative risk categories such as high, medium and low risk. The case study chemicals mostly fall within the same middle risk range of the matrix. Six chemicals fall into the higher exposure risk potential category and seven chemicals fall into the low exposure risk potential category based on the delineations shown in Figure 2. As a high tier screening, this type of representation may be useful for rapid visualization and categorization of large number of chemicals; however risk matrices should be used with caution when guiding risk management decisions [35].

Both the ranking and risk matrix approaches highlight the potential promise of multi-criteria decision analytic models for exposure-based prioritization, but further development beyond this effort is warranted. Given that the baseline weighting scenario – equal weights distributed among the chemical property and life cycle criteria – is likely an unrealistic one, a sensitivity analysis should be conducted to explore the effects of uncertainty in both the scoring of chemical parameters and the weighting schemes on the final chemical prioritization. This will help identify chemicals which are targets for further exposure assessment and data collection, ideally including better release characterization, proximal exposure assessment, and biomonitoring.

Finally, it is important to recognize that these results are strictly a measure of exposure potential and do not consider toxicological properties. Risk is a function of both hazard and exposure. The means by which organisms are exposed to stressors are complex; with many feedback loops (e.g., an outcome may itself become a stressor or modify other stressors). Risks related to chemical ingredients in products depend not only on the inherent properties of that chemical, but also the manner in which the chemical is formulated and used. Exposure potential therefore might be integrated with computational toxicology to paint a more complete picture of risk and to effectively prioritize the numerous chemicals in commerce.

### Conclusions

In this paper, we have presented a decision analytic approach to exposure-based prioritization of manufactured chemicals. The proposed methodology allows for structured and transparent analysis of chemical exposure potential through integration of heterogeneous metrics used to evaluate exposure risk-related information associated with both chemical properties and life cycle phases. The model is scalable to assess as many chemicals as is necessary for the project scope, and the MCDA framework is able to accommodate varied inputs and exposure potential indicators, providing an adaptive and easy-to-use screening tool for rapid prioritization in the face of sparse data. In addition, the use of weighting in the model allows for specific user objectives, expert judgment, and data availability considerations to be explicitly implemented within the assessment.

The proposed approach builds on earlier models and current research relating to rapid evaluation of exposure potential. Specifically, it integrates the results of mechanistic and statistical approaches with semi-quantitative categorical data to describe exposure potential. In this paper, we attempt to address the need for high-level screening tools that (1) are capable of more detailed assessments than those provided by simpler predictive models (i.e., limited to persistence and bioaccumulation as indicators of exposure), and (2) have less intensive data requirements than more complex models, so as to remain efficient at the screening level.

It is important to note that work on this model is ongoing, and that the initial framework presented in this paper is primarily intended to illustrate the application of decision analytic methods to supplement existing exposure potential estimation techniques. Currently, our developmental efforts are focused on: (1) refining ADME assessment criteria and calculations; (2) identifying optimal surrogates for bioaccumulation potential; (3) implementing value of information (VOI) techniques to quantify data gaps and prioritize further research efforts; (4) improving normalization algorithms; and (5) developing a supplemental logic model for more specific exposure scenario evaluation. Additionally, we are working to develop formal means of considering expert judgment and empirical chemical exposure data within our assessments. In the future, we anticipate that the decision analytic approach will be able to provide decision makers with important and reliable information to support efficient, exposure-based prioritization of manufactured chemicals.

## Supporting Information

File S1 Case Study Data.
(XLSX)
